# Unusual Postpartum Sacroiliitis Due to Burkholderia contaminans: Diagnostic Considerations From a Single Case

**DOI:** 10.7759/cureus.95401

**Published:** 2025-10-25

**Authors:** Ana S Montenegro Núñez, Carlos A Quezada, Daphne M Martinez, Eduardo G Arathoon, Rita A Pineda

**Affiliations:** 1 Internal Medicine, Universidad Francisco Marroquin, Guatemala, GTM; 2 Infectious Disease, Hospital Herrera Llerandi, Guatemala, GTM; 3 Rheumatology, Hospital Herrera Llerandi, Guatemala, GTM

**Keywords:** burkholderia cepacia complex, burkholderia contaminans, culture based therapy, infectious sacroiliitis, postpartum complications

## Abstract

Infectious sacroiliitis is a rare and often misdiagnosed condition, particularly in postpartum women, where symptoms may mimic benign musculoskeletal pain.

We describe a postpartum woman who developed severe lumbar and gluteal pain four days after a cesarean section. Examination revealed sacroiliac joint tenderness, positive FABER and Gaenslen tests, and mild asymmetrical lower extremity weakness. MRI demonstrated left-sided sacroiliitis with periarticular edema. Blood and synovial fluid cultures confirmed *Burkholderia contaminans*, an unusual finding in this clinical setting, which guided targeted therapy with trimethoprim-sulfamethoxazole. The patient completed intravenous followed by oral treatment, resulting in rapid pain relief, normalization of inflammatory markers, and complete recovery.

This case highlights the diagnostic challenge of sacroiliitis in postpartum women, where inflammatory marker elevation may be modest. It underscores the importance of a focused sacroiliac joint examination, culture confirmation, and susceptibility-guided therapy, while reminding clinicians to consider unusual pathogens when standard organisms are not isolated.

## Introduction

Infectious sacroiliitis (ISI) is a rare entity, accounting for only 1-2% of all septic arthritis cases [1]. It is frequently underdiagnosed due to its nonspecific symptoms and overlap with common causes of postpartum musculoskeletal pain. Pregnancy and the postpartum period confer increased susceptibility to ISI due to mechanical stress on the sacroiliac joint, hormonal effects on pelvic ligaments, and transient immunosuppression [2].

*Staphylococcus aureus *is the most frequently isolated pathogen, while *Streptococcus spp.*, *Escherichia coli*, and *Brucella spp.* are less common causes [3,4]. Diagnosis relies on imaging - particularly magnetic resonance imaging (MRI) - and microbiological confirmation.

*Burkholderia contaminans* (*B. contaminans*), a member of the *Burkholderia cepacia complex* (Bcc), is an opportunistic Gram-negative bacillus associated with contaminated pharmaceutical products and healthcare-associated infections, particularly in immunocompromised hosts [5]. To our knowledge, there are no previous reports of* B. contaminans* causing ISI.

We describe a postpartum woman who developed sacroiliitis due to this unusual pathogen, highlighting both the diagnostic challenges and the importance of considering atypical organisms in the differential diagnosis.

## Case presentation

A woman in her 30s with hypothyroidism and prior gestational diabetes presented with a two-week history of lumbar and gluteal pain that began four days after a cesarean section performed under epidural anesthesia. Postoperatively, she received ceftriaxone for 48 hours, followed by cefixime at home. Initially, the pain was mild (3/10 on the Numeric Rating Scale for pain [6]), localized to the upper medial quadrant of her left buttock, and partially relieved with nonsteroidal anti-inflammatory drugs (NSAIDs). However, symptoms worsened progressively, reaching severe intensity (10/10), prompting an emergency room visit.

On arrival, vital signs were stable. The patient was unable to bear weight due to pain. Passive movement of the left hip, including flexion and internal/external rotation, reproduced her pain. On palpation, there was marked tenderness localized to the posterior sacroiliac joint. Provocative maneuvers were positive, including FABER (Flexion, Abduction, External Rotation) and Gaenslen tests [7], and mild asymmetrical lower extremity weakness. The weakness was likely secondary to pain inhibition and periarticular inflammation, rather than direct neurological compromise, as no focal neurological deficits were documented.

Laboratory evaluation showed elevated inflammatory markers, including C-reactive protein (CRP) 29.7 mg/L and erythrocyte sedimentation rate (ESR) 22 mm/h, along with mild neutrophilia (Table 1). Blood cultures obtained at presentation remained negative.

**Table 1:** Laboratory results upon admission BUN: blood urea nitrogen; CRP: C-reactive protein; ESR: erythrocyte sedimentation rate

Parameter	Result	Reference range
White blood cells	8.59 × 10⁹/L	5.0–10.0 × 10⁹/L
Neutrophils	74%	40–60%
Lymphocytes	20%	20–45%
Hemoglobin	12.4 g/dL	12–16 g/dL
Hematocrit	49.2%	36–48%
Platelets	313 × 10⁹/L	150–500 × 10⁹/L
ESR	22 mm/h	0–20 mm/h
CRP	29.7 mg/L	0–5 mg/L
Creatinine	69.8 µmol/L	45–90 µmol/L
BUN	7.1 mmol/L	2.5–6.7 mmol/L

**Figure 1:**
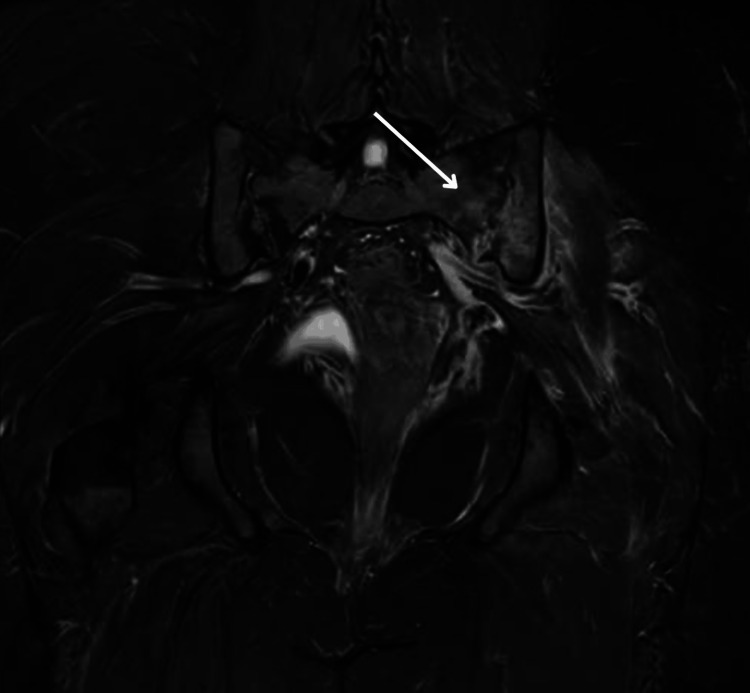
Coronal T2-weighted MRI of the pelvis Coronal T2-weighted sequence demonstrating increased signal intensity and periarticular edema involving the left sacroiliac joint (arrow), consistent with infectious sacroiliitis. The right sacroiliac joint appears preserved, highlighting the unilateral nature of the inflammatory process.

**Figure 2:**
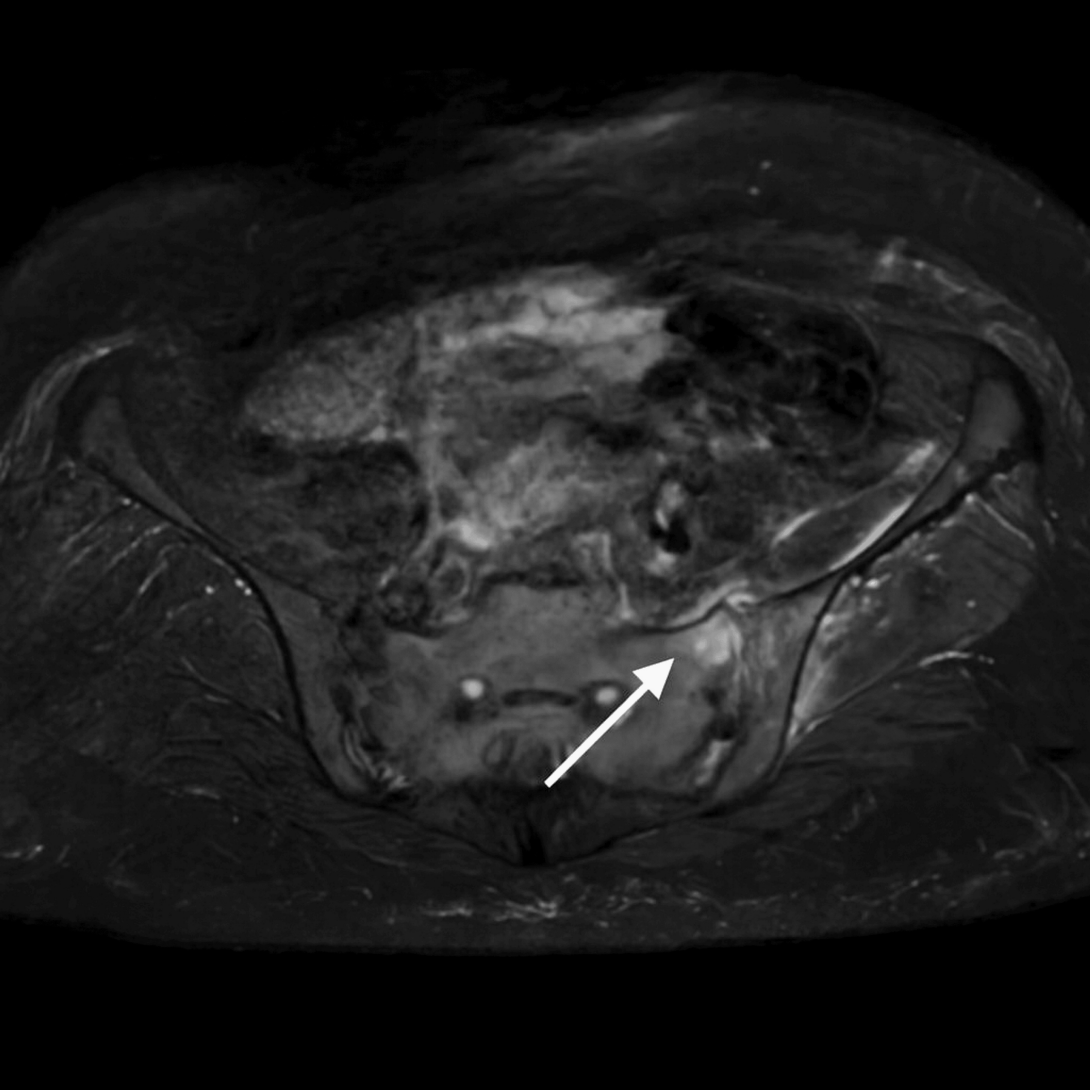
Sagittal T2-weighted MRI. Increased STIR signal in the subchondral bone of the left sacroiliac joint (arrow), confirming sacroiliitis.

Given the positive sacroiliac provocation tests and focal tenderness, an MRI of the pelvis was performed. Coronal T2-weighted sequences demonstrated increased signal intensity in the subchondral bone of the left sacroiliac joint, consistent with an inflammation. There was distension of the anterior synovial capsule with adjacent edema involving the iliacus and gluteus minimus muscles. Post-contrast images confirmed these inflammatory changes, consistent with active sacroiliitis (Figure 1 and Figure 2).

Differential diagnosis included postpartum mechanical back pain, sciatica and spondyloarthropathies. Given the unilateral involvement of the sacroiliac joint, an infectious etiology was strongly suspected. Blood cultures, synovial culture fluid aspiration and biopsies of the iliac and sacral bones were performed. Empiric intravenous vancomycin and ertapenem were started; however, pain persisted and CRP levels continued to rise.

Cultures from blood and synovial fluids yielded *B. contaminans*. Antimicrobial susceptibility testing demonstrated sensitivity to trimethoprim-sulfamethoxazole and levofloxacin. Based on these results, empiric antibiotics were discontinued, and intravenous trimethoprim-sulfamethoxazole (80/400 mg every 8 hours) was initiated. Pain improved rapidly, and the patient resumed ambulation with physical therapy. CRP levels declined steadily.

She was discharged after nine days with a peripherally inserted central catheter for 15 additional days of IV therapy, followed by oral trimethoprim-sulfamethoxazole. At follow-up, inflammatory markers had normalized, and pain had completely resolved, allowing independent mobility and return to childcare activities.

## Discussion

ISI is an uncommon condition, accounting for only 1-4% of all bone and joint infections, with a higher incidence observed in children and young adults [3,4]. *Staphylococcus aureus* is the most commonly isolated pathogen, although other organisms such as *Streptococcus spp.*, *Escherichia coli*, *Salmonella spp.*, *Brucella spp.*, and *Mycobacterium tuberculosis* have also been reported [3,8]. Risk factors for ISI include trauma, immunosuppression, rheumatoid arthritis, intravenous drug use, and pregnancy; however, up to 41% of cases occur without any identifiable risk factors [8]. In our patient, pregnancy was a contributing factor. ISI typically arises from hematogenous spread, most often from cutaneous, respiratory, or genitourinary sources. Less commonly, contiguous extension from adjacent infections, such as osteomyelitis, abscesses, or cellulitis may occur [8]. The relatively low vascularity of the sacroiliac joint reduces susceptibility to hematogenous spread, contributing to the condition’s rarity.

Pregnant and postpartum women account for 3.4-12.8% of ISI cases [9]. Susceptibility is linked to pregnancy-related mechanical stress, hormonal changes altering pelvic conformation, and transient immunosuppression. During childbirth, stretching or tearing of the sacroiliac ligaments and capsule can lead to synovitis or hemorrhage, which may present as postpartum lower back and buttock pain [2]. Because such symptoms are common, diagnosis of ISI is often delayed.

Clinical presentation is variable, and ISI is frequently misdiagnosed as mechanical back pain or sciatica. The absence of fever, reported in up to one-third of cases, may further obscure recognition [8]. Pain is usually unilateral, often left sided, and may radiate to the thigh or pelvis [10]. In our patient, the absence of systemic symptoms but presence of localized sacroiliac joint tenderness and positive provocation tests supported the suspicion of ISI.

Diagnosis relies on a combination of clinical, laboratory, microbiological and imaging findings. Laboratory markers are often nonspecific; leukocytosis and elevated inflammatory markers such as ESR and CRP are common, but not universal [8,11]. Studies indicate that CRP levels tend to be significantly higher in ISI patients compared to those with spondyloarthritis, supporting its diagnostic value [9]. In our case, inflammatory markers were only modestly raised (CRP 29.7 mg/L and ESR 22 mm/h). This may reflect either the organism's virulence profile or a suppressed inflammatory response in the postpartum setting.

Imaging is central to diagnosis, with MRI regarded as the gold standard due to its superior sensitivity over CT and bone scintigraphy [4,8,11]. Characteristic MRI features include T2 hyperintense signals, bone marrow edema, and periarticular contrast enhancement [8,9]. Kang et al. demonstrated that MRI is both highly specific for inflammatory sacroiliitis and highly sensitive for infectious cases. Findings that favor infection include capsulitis, extracapsular fluid collections, and periarticular muscle edema [12]. In our patient, enhancement of the articular capsule with periarticular edema aligned with these reported features, confirming the diagnosis.

Although bone scintigraphy can demonstrate sacroiliac joint infection, its diagnostic accuracy is lower than that of MRI (likelihood ratio 3.4 versus 10) [12]. CT can provide complementary information, but is often normal in the early disease, as reported in up to 22.4% of cases [13]. Similarly, radiographs are rarely useful in early detection, as abnormalities usually appear late in the course of infection [11,12].

Microbiological confirmation is essential, although cultures are positive in only two-thirds of cases [11,12]. Empiric therapy is usually directed against *S. aureus*, with subsequent tailoring based on susceptibility results [9,11]. No standardized antibiotic duration exists, but a typical regimen involves at least two weeks of intravenous therapy, followed by four to six weeks of oral treatment. In refractory cases or those complicated by abscess, surgical drainage may be required [8].

Our case is notable for the isolation of* B. contaminans*, a member of the Bcc. Organisms within the Bcc are aerobic, Gram-negative bacilli recognized as opportunistic pathogens in healthcare settings, frequently linked to contamination of medical products and multidrug resistance [14,15]. Their ability to survive in aqueous environments, form biofilms and resist disinfectants poses infection-control challenges [5]. Antimicrobial susceptibility varies among Bcc species, though some strains remain sensitive to trimethoprim-sulfamethoxazole, ceftazidime, and fluoroquinolones [16]. Rising resistance has prompted investigation into novel agents, including glycopolymer-based therapies [17]. In this patient, the isolate was sensitive to trimethoprim-sulfamethoxazole and levofloxacin, which is noteworthy given the multidrug-resistant profile often associated with the Bcc. This susceptibility allowed effective stepwise intravenous-to-oral therapy.

Outbreaks of *B. contaminans* have been associated with contaminated hospital equipment, intravenous solutions, and medications [18,19]. In the context of a recent cesarean section, potential sources include anesthetic or perioperative fluids. Although a definitive source was not identified, the strong association of *Burkholderia* species with contaminated medical products underscores the importance of strict infection-control protocols in perioperative care.

Finally, the mild asymmetrical weakness observed in our patient was most consistent with pain inhibition rather than neurological compromise. The absence of focal neurological deficits or radiological evidence helped rule out alternative diagnoses such as epidural abscess.

## Conclusions

This case highlights the diagnostic challenges of ISI in postpartum women, a population in whom musculoskeletal pain is common and often underestimated. The unusual isolation of *B. contaminans* guided a tailored antimicrobial strategy, and early MRI facilitated prompt diagnosis. Culture-directed therapy with trimethoprim-sulfamethoxazole led to complete recovery. Importantly, this case emphasizes the need for strict infection-control measures in obstetric and perioperative care, given the recognized association of *B. contaminans* with contaminated medical products.
